# Characterization of Two BAHD Acetyltransferases Highly Expressed in the Flowers of *Jasminum sambac* (L.) Aiton

**DOI:** 10.3390/plants11010013

**Published:** 2021-12-21

**Authors:** Yuting Wang, Hongliang Zhang, Chao Wan, Xian He, Jinfeng Huang, Meiling Lyu, Yuan Yuan, Binghua Wu

**Affiliations:** 1 Ornamental Horticulture Department, College of Horticulture, Fujian Agriculture and Forestry University, No. 15, Shang Xia Dian Road, Cangshan District, Fuzhou 350002, China; yutingwang123456@163.com (Y.W.); xihengdesign@163.com (H.Z.); 1190371007@fafu.edu.cn (C.W.); strings_he5@sina.com (X.H.); 1190371002@fafu.edu.cn (J.H.); mllyu@fafu.edu.cn (M.L.); yuanyuan@fafu.edu.cn (Y.Y.); 2 The Fujian Provincial Key Laboratory of Plant Functional Biology, College of Life Sciences, Fujian Agriculture and Forestry University, No. 15, Shang Xia Dian Road, Cangshan District, Fuzhou 350002, China

**Keywords:** *Jasminum sambac*, Oleaceae, BAHD acyltransferases family, benzyl alcohol O-acetyltransferase (BEAT), enzyme kinetics, floral scent, benzenoid, benzyl acetate

## Abstract

Volatile benzenoid compounds are found in diverse aromatic bouquets emitted by most moth-pollinated flowers. The night-blooming *Jasminum sambac* is widely cultivated worldwide in the tropics and subtropics for ornamental and industrial purposes owing to its fragrant flowers. Benzylacetate is a characteristic constituent in jasmine scent which makes up to approximately 20–30% of the total emission in the headspace or extract, but the biosynthesis enzymes and the encoding genes have not yet been described. Here, we identify two cytosolic BAHD acyltransferases specifically expressed in the petals with a positive correlation closely to the emission pattern of the volatile benzenoids. Both JsBEAT1 and JsBEAT2 could use benzylalcohol and acetate-CoA as substrates to make benzylacetate in vitro. The recombinant GST-JsBEAT1 has an estimated apparent *K*_m_ of 447.3 μM for benzylalcohol and 546.0 μM for acetate-CoA, whereas in the instance of the His-JsBEAT2, the *K*_m_ values are marginally lower, being 278.7 and 317.3 μM, respectively. However, the catalytic reactions by the GST-JsBEAT1 are more efficient than that by the His-JsBEAT2, based on the steady-state *k*_cat_ parameters. Furthermore, ectopic expression of JsBEAT1 and JsBEAT2 in the transgenic *P. hybrida* plants, driven by a flower-specific promotor, significantly enhances the biosynthesis of benzylbenzoate and benzylacetate, as well as the total VOCs.

## 1. Introduction

Plants utilize a wide array of specialized metabolites to interact with changing environments and to safeguard their survival and reproduction [1,2,3]. These structurally different molecules produced by plants are usually classified based on their biosynthetic origin, such as terpenoids, benzenoids/phenylpropanoids, fatty acid derivatives and amino acid derivatives. Large portions of the plant-specialized metabolites are lipophilic small molecules with high vapor pressure at ambient temperatures, and thus called volatile organic compounds (VOCs). More than 1700 VOCs have been identified from 90 different plant families, and mostly are synthesized and released, with high abundance and diversity, from the flowers in many flowering plants [4]. Floral VOCs play important roles in the attraction of pollinators and protection against pathogens, parasites, and herbivores [5,6,7].

Flowers of the night-blooming plant *Jasminum sambac* (L.) Aiton (Oleacese) emit a strong fragrance which has long been used as raw material in the perfume industry and in scented tea. While jasmine absolute is the common name of the aroma product made by distillation from subsequent hexane and ethanol extractions of the flowers, the VOCs are enriched in benzyl acetate, linalool and (E,E)-α-Farnesene [8]. Floral VOCs in several *Jasminum* species have also been reported [9,10,11,12,13,14]. Although variation in the composition of the floral scent exists depending on the species/varieties, cultivation practices, and season, benzyl acetate is the predominant benzenoid ester, accounting for almost 90% of the volatile benzenoid/phenylpropanoids. However, the biosynthetic enzymes and the encoding genes are rarely explored in this genus.

Phytochemically, most floral benzenoid esters are produced by acyl-CoA-dependent acylation performed by a group of acyltransferases, the Benzylalcohol *O*-acetyl-, Anthocyanin *O*-hydroxycinnamoyl-, Hydroxycinnamoyl/benzoyl-CoA: Anthranilate *N*-hydroxycinnamoyl/benzoyl-, and Deacetylvindoline 4-*O*- acetyltransferases (BAHD) superfamily [15,16,17,18]. These enzymes catalyze the transfer of an acyl group from activated acyl donor molecules, Coenzyme A or CoA thioesters, to acyl acceptor molecules, oxygen- and nitrogen-containing substrates, producing esters and amides, respectively [19]. A benzyl–alcohol acetyl-CoA acetyltransferase (BEAT) from *Clarkia breweri* involved in floral VOC synthesis was first identified and cloned in 1998 [17]. Since then, more than 69 biochemically characterized BAHD acyltransferases have been reported, and they share two conserved motifs, HXXXD and DFGWG, but with low overall sequence identity at the amino acid level of 25–34% [19,20]. Phylogenetically, the plant BAHD family could be divided into five clades [15] or further refined to eight clades [21]. The versatile reactions catalyzed by these divergent enzymes make it difficult to predict their function solely based on the encoding sequences.

In this study, two highly expressed and volatile benzenoid profile-correlated candidate *BAHD* genes are identified and characterized from *J. sambac* petals. The enzymatic activities of the recombinant proteins were determined using common substrates benzyl–alcohol and acetyl-CoA. Finally, transgenic *P. hybrida* plants ectopically expressing these two *J. sambac* genes in the flowers showed enhanced production of the major benzenoid volatiles, suggesting that both are functional *BAHD* in planta. Our results provide molecular and biochemical evidence for further deciphering the genetic control of volatile benzenoid production in the flowers of *J. sambac*.

## 2. Results

### 2.1. Profiling Floral Scent Volatiles in J. sambac

**Figure 1:**
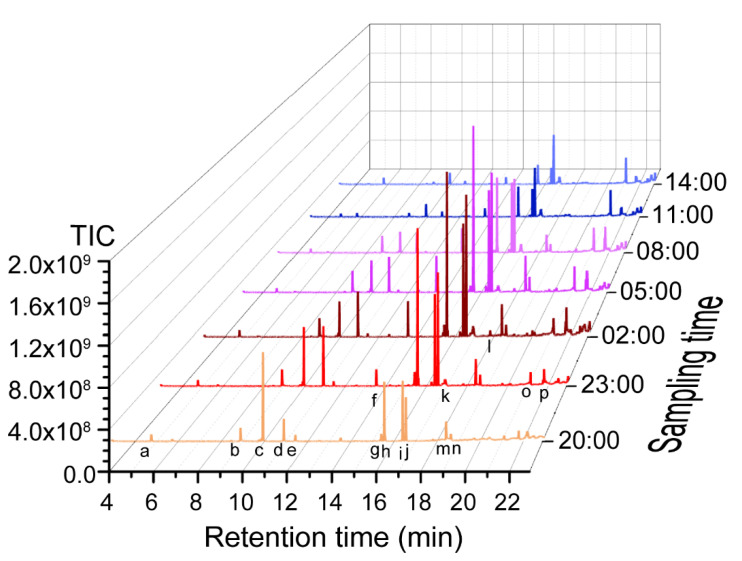
Floral volatile profiling during flowering lifetime. *J. sambac* flowers were sampled at a 3 h interval after opening at the evening and the methanol extracts of petals were subjected to GC-MS analysis. The experiment was conducted using at least three sets of samples representing 24 pots of plants. Shown here is one set of results. Except for benzyl alcohol (RT 8.89 min, b), linalool (RT 9.93 min, c), benzyl acetate (RT 10.90 min, d), and α-farnesene (RT 15.55 min, h), peaks were tentatively identified based on RI and mass spectrum (see Figure S1): a, 3-hexenal; b, benzyl alcohol; c, linalool; d, benzyl acetate; e, methyl salicylate; f, methyl anthranilate; g, germacrene D; h, α-farnesene; i, cis-hexenyl benzoate; j, germacrene D-4-ol; k, 1-docosene; l, unknow; m, benzoic acid-2-propenyl ester; n, benzyl benzoate; o, nerolidol; p, methyl linolenate.

The flower of *J. sambac* starts to emit fragrance when it opens at dusk. We sampled the open flowers at 3 h intervals and determined the volatile profiles using methanol extracts via a GC-MS. The total volatiles peaked at around midnight and featured large parts of terpenoids and benzenoids (Figure 1). The peak-corresponding compounds were putatively identified based on the RI (Kovats retention indices) and MS spectrum matching the NIST (National Institute of Standards and Technology) GC-MS databases (Figure S1), and authentic reference substance of α-farnesene, benzyl acetate, benzyl alcohol, and (R/S)-linalool were also used for the confirmation. The abundant constituents were α-farnesene, linalool, germacrene, benzyl acetate, and other benzoic acid esters (Figure 1), consistent with data reported in earlier literature [10,13].

We quantified the benzenoid compounds in the petal extracts using an internal standard as a reference and found that cis-3-hexenylbenzoate and benzyl acetate were the two most enriched compounds, followed by methylanthranilate and benzoic acid-2-propenyl ester at about the midnight timepoints (Table 1). Thus, the petal extract was characterized of a mixture of benzenoid esters with some variations during the time.

### 2.2. Cloning of Petal-Specific BAHD-Like cDNAs

We sought to identify the benzyl alcohol acyltransferase genes that were specifically expressed in the petal. By BLAST searching the floral transcriptome [22] using sequences of known proteins from *C. breweri* [23], *P. hybrida* [24], and *Populus trichocarpa* [25] as the query, we found at least 18 homologous EST sequences expressed in the petal (Figure S2). After removing shorter (<500 bp) or redundant sequences, the unigenes were matched to full-length cDNAs in a lab-generated PacBio SMRT long-read transcriptome dataset via BLAST search, finally yielding seven unique BAHD-like homologs (Figure S2). These genes were designed as *JsBEAT1* through *seven* and the deduced amino acid sequences were used to align with known enzymes from other species (Figure S3). The *JsBEAT1-*, *2-*, *4-*, and *5*-encoded proteins belong to the Clade V, whereas *JsBEAT3* and *6* code for Clade IV enzymes, and the *JsBEAT7* protein clusters into Clade III (Figure S3). The two conserved motifs HXXXD and DFGWG of BAHD acyltransferases are presented in the seven JsBEAT proteins, although the DFGWG motif in JsBEAT3 and 6 have a substituted Cys and Gly for the Trp, respectively. Moreover, the negative Asp residue at the DFGWG motif in JsBEAT2 is replaced by a highly polar Asn (Figure S4).

**Figure 2:**
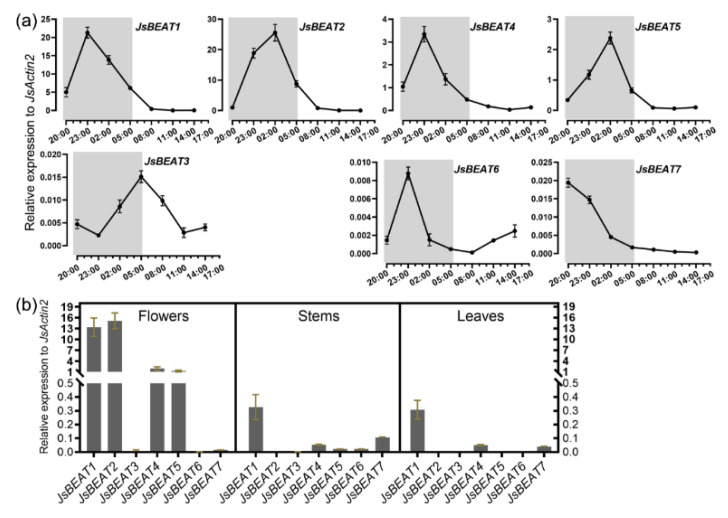
Expression profiles of seven floral-specific BAHD-like genes detected via quantitative RT-PCR. (**a**) Time-resolved transcript level in petals post anthesis. Shaded area indicates the night period; (**b**) transcript abundance in the tissues. The flower samples were taken at ~21:00 h. The error bars represent SD of three biological replications (sets of 10 flowers from 5 different plants). Each qRT-PCR reaction consisted of at least 3 technical repeats.

We further determined the transcript abundance of the seven *JsBEAT* genes in the opening petal and in different tissues using quantitative RT-PCR. In the petal, the expression of *JsBEAT1*, *4*, *6*, and *7* peaked earlier at night, with the maximum at about 23:00 h, while *JsBEAT2*, *3*, and *5* showed delayed expression peaks by ~3–6 h (Figure 2A). Among the genes, *JsBEAT2* and *1* were the most expressed and their transcript levels were approximately 7–11 folds of that of the moderately expressed *JsBEAT4* and *5* (Figure 2B). The other three genes, *JsBEAT3*, *6* and *7* were expressed with minimum or hardly detectable level in all tissues tested (Figure 2). Interestingly, *JsBEAT1* was also the most expressed among the seven genes in stems and leaves, though at a relatively lower level (Figure 2B). It seems that the petals of *J. sambac* mainly express the Clade V BAHD acyltransferase genes, *JsBEAT1*, *2*, *4*, and *5*.

The subcellular localization of the two highly expressed JsBEAT1 and JsBEAT2 were also monitored using C-terminal GFP-tagged fusion transiently expressed in *J. sambac* petal protoplasts and in the leaf epidermis of *N. benthamiana*. It could be observed that both were localized to the cytosol (Figure S5). In addition, the JsBEAT1-GFP also exhibited strong fluorescent signals in the nucleus whereas the JsBEAT2-GFP displayed obvious localization in unknown intracellular structures, as seen much more in the heterologously expressing leaves (Figure S5). These results were roughly consistent with the predicted subcellular localization by three different programs (Table S1). The pIs of JsBEAT1 and JsBEAT2 proteins were predicted as 7.69 and 7.61, respectively. Given a normal pH of ~7.0–7.2 in the cytosol, both proteins would be positively charged when residing in the cytoplasm.

### 2.3. Enzymatic Characterization of the Recombinant JsBEAT1 and JsBEAT2

We then expressed the full-length cDNAs in *E. coli* to obtain purified recombinant proteins, using a N-terminal fusion of 6xHis-tag or a GST-tag. The recombinant 6xHis-JsBEAT2 was readily generated; however, the JsBEAT1 was only successfully produced as a GST-tagged fusion (Figure S6).

Although purified recombinant proteins contained either the 6xHis-or the GST-tag at the N-termina, they did show catalytic activities converting the substrates benzyl alcohol and acetyl-CoA to benzyl acetate in a preliminary experiment. Thus, the recombinant proteins were used in serial reactions with different substrate concentrations to determine the enzymatic property.

**Figure 3:**
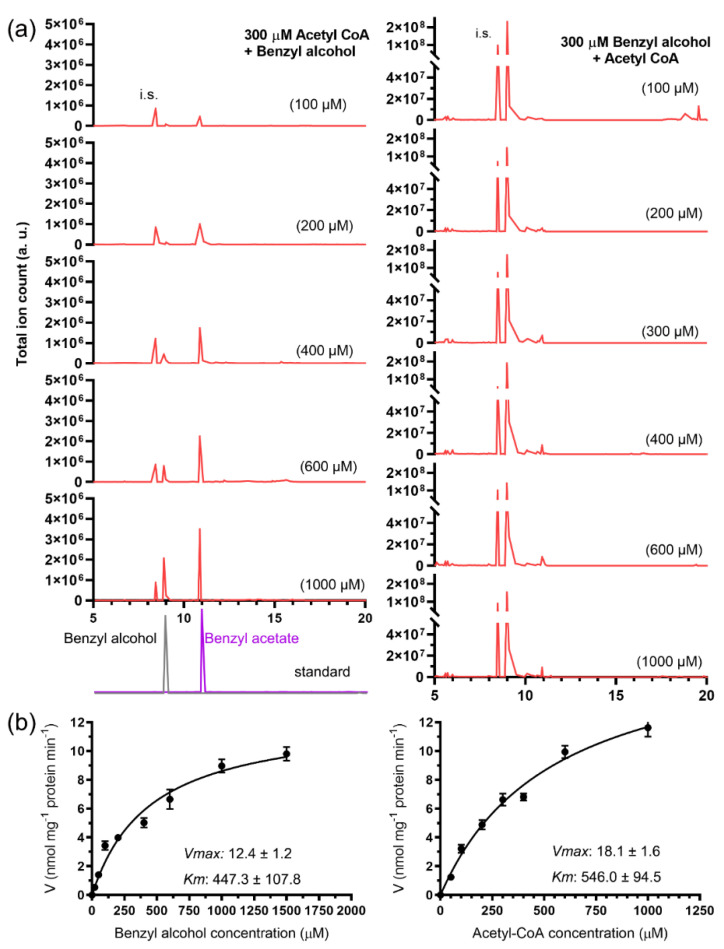
The recombinant GST-JsBEAT1 catalyzed the in vitro formation of benzyl acetate from benzyl alcohol and acetyl-CoA. (**a**) Representative chromograms of the reaction products; (**b**) curve fitting against the two substrates, *R*^2^ = 0.97 and 0.99, respectively. The experiments were repeated at least three times using three sets of recombinant proteins.

Using 300 μM acetyl-CoA as the acyl doner, the apparent *V*max and *K*m of the GST-JsBEAT1 for substrate benzyl alcohol were estimated as 12.4 ± 1.2 nmol mg^−1^ protein min^−1^ and 447.3 ± 107.8 μM, respectively. At a fixed 300 μM benzyl alcohol as the acceptor, the estimated apparent *V*max and *K*m of the GST-JsBEAT1 against the acetyl-CoA was 18.1 ± 1.6 nmol mg^−1^ protein min^−1^ and 546.0 ± 94.5 μM, respectively (Figure 3). Using 1 μM fixed concentrations of substrates, similar results were obtained. Under these conditions, we calculated the turnover number *K*cat of the GST-JsBEAT1 as 201.5 ± 4.1 s^−1^ benzyl alcohol or 316.8 ± 4.4 s^−1^ acetyl-CoA.

**Figure 4:**
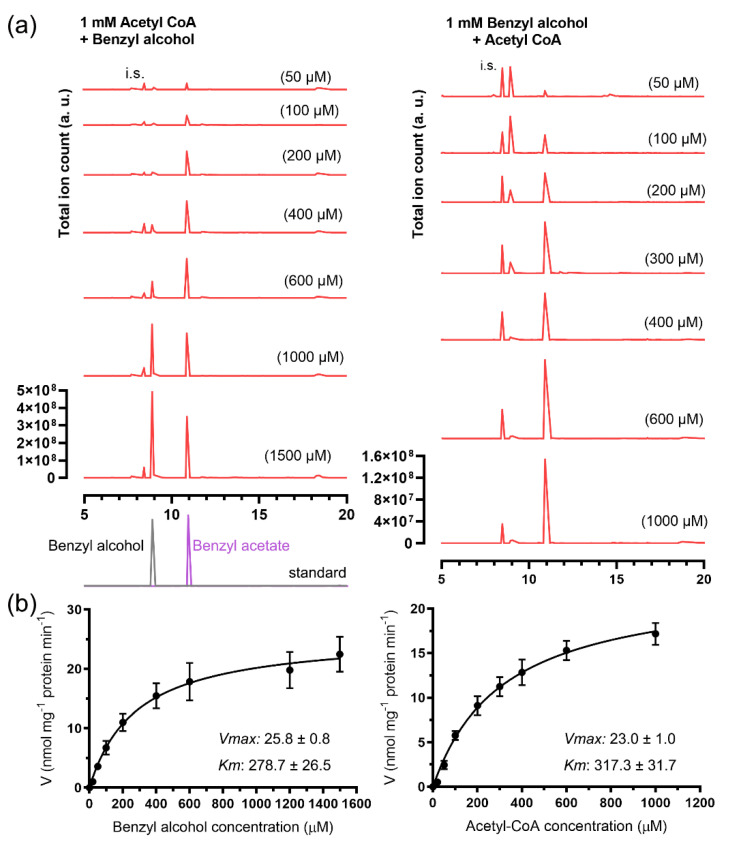
The recombinant 6xHis-JsBEAT2 catalyzed the in vitro formation of benzyl acetate from benzyl alcohol and acetyl-CoA. (**a**) Representative chromograms of the reaction products; (**b**) curve fitting against the two substrates, *R*^2^ = 0.99 and 0.99, respectively. The experiments were repeated at least three times using three sets of recombinant proteins.

The recombinant 6 × His-JsBEAT2 showed slightly higher activities to catalyze the formation of benzyl acetate than the GST-JsBEAT1. Its *V*_max_ and *K*_m_ for the substrate benzyl alcohol were 25.8 ± 0.8 nmol mg^−1^ protein min^−1^ and 278.7 ± 26.5 μM, respectively. For the substrate acetyl-CoA, a *V*_max_ of 23.0 ± 1.0 nmol mg^−1^ protein min^−1^ and a *K*_m_ of 317.3 ± 31.7 μM were estimated (Figure 4). However, the 6xHis-JsBEAT2 had a lower *k*_cat_ for benzyl alcohol (36.8 ± 0.6 s^−1^) and for acetyl-CoA (25.7 ± 0.4 s^−1^) as well as the GST-JsBEAT1.

Taken together, these in vitro assays indicated that both JsBEAT1 and JsBEAT2 were able to catalyze the acryl transfer from acetyl-CoA to benzyl alcohol and produce benzyl acetate. By comparison, JsBEAT2 seemed to be slightly higher in affinity to both substrates since its *K*_m_ values for both were lower by a factor of ~0.6. However, the enzyme reaction by JsBEAT1 was more efficient (Table 2).

### 2.4. Ectopic Expression of JsBEAT1 and JsBEAT2 in Flowers of Transgenic Petunia hybrida Induced More Benzylbenzoate and Benzylacetate

To assess the function of the two JsBEAT enzymes *in planta*, we generated transgenic plants ectopically expressing *JsBEAT1* and *JsBEAT2* in *P. hybrida* ‘Mitchell Diploid’ (W115) because an attempt to transform *J. sambac* had not been successful. The *JsBEATs* were expressed as a *GFP* fusion driven by 1 Kb promoter fragment of the flower-specific *C. breweri linalool synthase* (*LIS*) [26,27]. We obtained T2 transgenic plants by subsequent screening of seed germination on kanamycin-containing medium. The transgenic plants showed normal flowering phenotype but developed slightly more flowers per plant (Figure S7a). The T-DNA insertion was verified by using PCR amplification (Figure S7b), and the mRNA level of the transgenes was measured as one order of magnitude over the wild-type W115 background via qRT-PCR with most independent lines (Figure S7c). The expression of the fusion proteins was further confirmed by Western blotting using an anti-GFP antibody (Figure S7d). In addition, we also observed the GFP fluorescent signal in the epidermic cells of the corolla from transgenic plants, which showed localization to some unknown granule subcellular structures and was different to those observed above in the transiently transfected *J. sambac* petal protoplasts and the *N. benthamiana* leaves (Figure S7e).

The flowers of the *P. hybrida* W115 emit mainly benzenoid and phenylpropanoid volatiles derived from three metabolic pathways of phenylalanine [24,28,29,30]. Using hexanol extraction, we determined the volatile compounds in the petals of the W115 and the transgenic plants via a GC-MS (Figure S8). The compounds were tentatively identified based on their Kovats retention index (RI) and MS spectrum comparison with known compounds in the NIST MS database (Figure S9).

**Figure 5:**
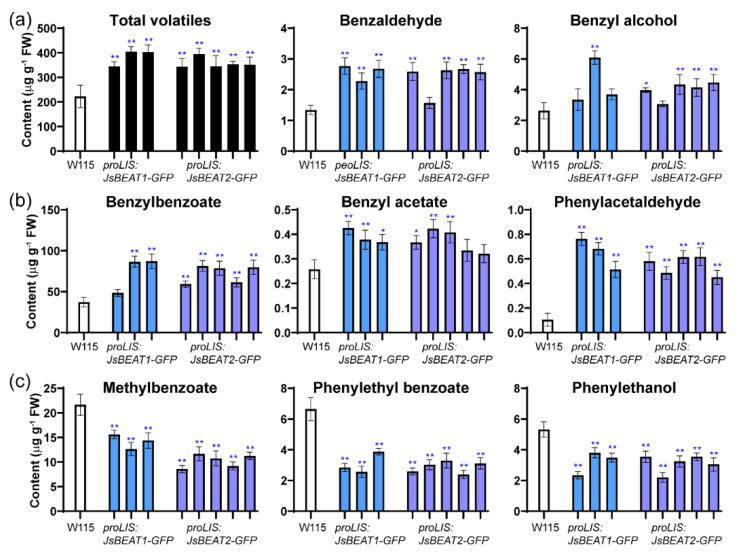
Floral expression of *JsBEAT1* and *JsBEAT2* in transgenic *P. hybrida* W115 enhances major benzenoid volatile production but decreased methyl benzoate and phenylethyl benzoate. (**a**) and (**b**) show the increased compounds and the total amount of compounds, while (**c**) shows the reduced compounds. The quantification is relative to an internal standard isobutylbenzene. Error bars represent SD of three independent assays on pooled samples from 5–7 flower. Significant level: * *p* < 0.05; ** *p* < 0.01 confirmed by Student’s *t*-test.

**Figure 6:**
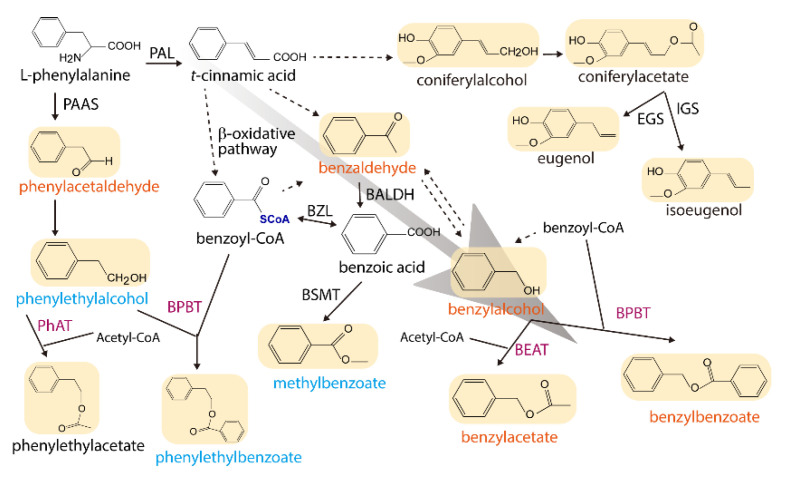
A simplified metabolic network for major floral volatile benzenoid/phenylpropanoid compounds in *P. hybrida* W115, depicting the effects of ectopic expression of the two *JsBEATs*. The volatile compounds are shades and the names in tangerine orange, cyan blue, and black indicate the compound is increased, decreased and no change or not detected, respectively. Solid arrow indicates a described conversion while a broken arrow denotes multiple steps and/or uncharacterized reactions. Enzyme abbreviations: BALDH, benzaldehyde dehydrogenase; BEAT, benzylalcohol acetyl-CoA acetyltransferase; BPBT, benzylalcohol/phenylethanol benzoyl-CoA benzoyltransferase; BSMT, benzoic acid/salicylic acid carboxyl methyltransferase; BZL, benzoate:CoA ligase; EGS, eugenol synthase; IGS, isoeugenol synthase; PAAS, phenylacetaldehyde synthase; PAL, phenylalanine ammonia lyase; PhAT, phenylalcohol acetyl-CoA acetyltransferase. Enzymes in violet are members of the BAHD acyltransferase family. The background gray arrow highlights the volatile flux driven by the overexpression.

Quantification of the relative abundance of the volatile compounds indicated that major benzenoid and phenylpropanoid production in the flower was enhanced by the ectopic expression of both *JsBEAT* genes. The total content of volatiles in the corolla was increased by a factor of 1.5–1.9 compared to that in the W115 (Figure 5a). Notably, bezylbenzoate, the most abundant component of the floral volatiles, increased by almost 2-folds in most transgenic lines, while the minor compound benzylacetate by about 50% (Figure 5b). However, concentration of the other two benzoate acryl esters, methylbenzoate and phenylethylbenzoate, were declined to approximately one-half of that in the W115 (Figure 5c). Other volatiles, such as benzaldehyde, benzylalcohol, and phenylacetaldehyde were also accumulated in the transgenic flowers, whereas phenylethanol was reduced (Figure 5). Overall, no obvious phenotypical difference between the two transgenes was observed, and the overexpression of both genes significantly enhanced the synthetic flux to benzylbenzoate and benzylacetate, as well as a concomitant rise in benzylalcohol and benzaldehyde (Figure 6).

## 3. Discussion

After blooming at night, *J. sambac* flowers produce and emit various volatile benzenoid compounds among others, with benzylacetate occupying ~one-third to one-half of headspace volatiles and ~10 to 30% in the extracts (Table 1) [8,9,14]. In this study, we have identified at least four candidate BAHD acyltransferase-coding genes that were expressed preferentially at similar time window (Figure 2 and Figures S2–S4). Two highly expressed *JsBEAT1* and *JsBEAT2* were full-length cDNA cloned, and the encoded proteins could convert benzyl–alcohol to benzylacetate by using acetyl-CoA as the acyl donor in vitro. In addition, *JsBEAT4* and *5* were also moderately expressed (having a transcript abundance of ~one-seventh of *JsBEAT1* and *2*). Since we did not characterize these two genes, it could not be excluded that these two might also contribute to the formation of benzylacetate in the *J. sambac* flowers. Although not tested, both JsBEAT1 and JsBEAT2 may also be capable of making other minor acetylated scent compounds found in the *J. sambac* flowers, in addition to benzylacetate. The question remains as to what extent may individual JsBEAT contribute to the floral benzenoids production, which requires further experiments using gene knockout or other approaches.

At the amino acid level, JsBEAT1 shares a 48.86, 28.24, and 25.89% identity with JsBEAT4, JsBEAT2, and JsBEAT5, respectively, while JsBEAT2 is 27.40 to 32.22% identical to JsBEAT4 and JsBEAT5. Phylogenetically, the four petal-expressing acyltransferases are grouped into the clade V of the BAHD family (Figure S3). This clade includes some previously described members such as the benzyl alcohol/phenylethanol benzoyl-CoA benzoyltransferases (BPBT) from *P. hybrida* and *C. breweri* flowers and leaves, which are responsible for volatile benzenoids production via the β-oxidative benylbenzoate synthetic pathway [23,24]. Another example comes from the Concord grape; the VlAMAT catalyzes the formation of methyl anthranilate from anthraniloyl-CoA and methanol in the berry [31]. Additionally, other reported BAHD-clade V acyltransferases catalyze the formation of *O*- or *N*-acylation on diverse substrates with different acyl-CoA donors, and may be involved in defense against herbivores, UV-protection, and pathogen resistance, as well as in cell-wall biosynthesis [15,19]. Our results showed that the two JsBEAT could equally enhance the floral benzenoid volatile production, especially the accumulation of benzylbenzoate and benzylacetate (Figure 6) in the transgenic *P. hybrida* plants, suggesting that they may be functionally redundant in the *J. sambac* flowers. Or else they may possess a subtle, not-yet-determined catalytic preference depending on the substrate availability and subcellular compartmentalization. Indeed, the subcellular localizations of both JsBEAT1 and 2 in transgenic *P. hybrida* were found unexpectedly in granule cytosolic structures (Figure S6e), other than in the bulk cytoplasm [19] as seen in *Jasminum* petal protoplasts (Figure S5). The nature of the observed cellular granule structures is not known; however, peroxisomes or mitochondria or membrane vesicles are considered as possible candidates, as such the two enzymes may be recruited by specific unknown proteins from the heterologous *P. hybrida* flower tissue. These possibilities require further investigation.

The apparent *K*m for both acyl donor (acetyl-CoA, ranging from 317.3 to 546.0 μM) and acyl acceptor (benzyl alcohol, ranging from 278.7 to 447.3 μM) are rather high in our enzymatic assay using GST-tagged or 10 × His-tagged proteins, as compared to the canonical CbBEAT, CbBEBT and CbCHAT [23,32], indicating that both acetyl-CoA and benzyl alcohol may not be the preferred substrates for the two JsBEATs. The apparent *K*m for a preferred substrate in many plant BAHDs is reported ranging from several to two hundred μM ([33,34]). However, some BAHDs may exhibit higher *Km* (>300 μM for the preferred substrates) [35]. Thus, it will be necessary to further test the enzymatic activity of the two JsBEAT using different donor or acceptor substrates to determine the preferent substrates.

In summary, two highly expressed BAHD acyltransferases, capable of acylating benzylalcohol and other alcohol compounds, are likely responsible for the volatile benzenoid production in the nigh blooming fragrant flowers of *J. sambac*, with somewhat functional redundance.

## 4. Materials and Methods

### 4.1. Plants and Culture Conditions

Four-year-old *J. sambac* (L.) Aiton cv ‘shuangban’ plants were propagated from cuttings and maintained in the greenhouse. Plants were transferred to 30 cm containers in a climate room 6 months before the experiments, where the plants received LED illumination at ~245 μmol m^−2^ s^−1^ with a day/night regime of 16/8 h. The climate room was configured at a day/night temperature of 28/22 °C and a constant humidity of 70%. *P. hybrida* ‘Mitchell Diploid’ (W115) was seed propagated and grown in a climate room under similar conditions.

### 4.2. Volatile Extration and GC-MS Determination

Labeled flowers of *J. sambac* plants were collected at consecutive time points from fresh opening throughout senescence, starting at 20:00 h of the first day. The petals were separated, weighed, frozen in liquid nitrogen, and grounded into fine powders. About 400 mg powders were extracted with hexane (cat. Nr. H100107, Aladdin, Shanghai, China) and 1.11 μg mL^−1^ isobutylbenzene (cat. Nr. 113166, Merck, Darmstadt, Germany) was spike-in as an internal standard. After 3 h incubation at room temperature (RT) with rotation at 150 rpm, the supernatant was collected by centrifugation at 1300 rpm for 10 min and filtered through a 0.22 μm membrane (Merck, Darmstadt, Germany). The clear extracts were used for GC injections and 5 injections were conducted for each extract. Usually, 5 to 10 flowers from 9 plants were collected and pooled as one biological sample, which were repeated in three replications. After flowering, the plants were subjected to light pruning. The experiment was repeated once in the next flowering flush.

Sampling of *P. hybrida* W115 flowers was performed at 23:00 h of day 2 and 3 post opening when the emission was peaked [36]. Pooled corolla was used for hexane extraction as mentioned above.

A PerkinElmer Clarus 680 GC equipped with a SQ8 TGC/MS detector and an Agilent J&W HP-5MS capillary column (0.25 mm diameter, 30 m length, and 0.25 μm film thickness) was employed for the GC-MS analysis. Oven temperature program was set as: started at 40 °C for 2 min, then raised at 10 °C min^−1^ to 250 °C. The MS interface temperature (split injection) was 280 °C. MS EI scan range was 40 to 500 *m*/*z*. Peaks were calibrated using *n*-hydrocarbons (C8–C25, Sigma, Saint Louis, MO, USA) to convert the retention time of each component to Kovats Retention Index (RI) under the same conditions.

Volatile compounds were putatively identified based on their RI and MS spectra compared to those in the NIST11 MS library and when available, verified by authentic reference substances. To quantify the concentration of the compounds, the peak area was compared to an internal standard and calibrated further by using curves made with authentic references or closet hydrocarbons.

### 4.3. RNA Isolation, Real-Time RT-PCR, Full-Length cDNA Cloning, and Vector Construction

Total RNA was isolated using a TransZol Up Plus RNA Kit (TransGen Biotech, Shanghai, China). Synthesis of cDNA was conducted in a 20 μL reaction containing Oligo d(T)18 primers and the transcript^®^ RT/RI Enzyme Transcriptase (TransGen Biotech) using approximately 1 μg total RNA as templates.

Real-time RT-PCR was run on a LightCycler 96 (Roche Molecular Systems, Inc., Pleasanton, CA, USA) and each 20 μL reaction contained 1 μL cDNA, 10 μL of 2×TransStart^®^ Green qPCR SuperMix (TransGen Biotech, Shanghai, China), and 0.2 μM of each primer pair (Table S2). We verified the specificity and amplification efficiency of all primer pairs by using melt curves and five-point calibration of ten-fold serial dilutions. Quantification of each gene expression was determined at least by three technical repeats and three biological replications using two reference genes, *JsACTIN2* and *JsUBQ10*, which yielded comparable results.

The full-length *JsBEAT1* and *JsBEAT2* cDNA were PCR amplified using designed primers (Table S2) and cloned into the GATEWAY pENTR/D-TOPO vector, respectively. Subcloning into the binary vector pK7FWG2.0 [37] was achieved by using the LR reaction of the respective pENTR/D-TOPO plasmid harboring the respective JsBEAT. The generated pK7-JsBEAT1 and pK7-JsBEAT2 were used for PEG-mediated protoplast transformation and *Agrobacterium* *tumefaciens* mediated leaf infiltration. These two vectors utilized the Cauliflower Mosaic Virus 35S promoter to drive the expression.

To construct a floral-specific version of binary vector, we first synthesized a 1031 bp fragment of the 5′-sequence of the *C. breweri* LIS gene [27] with incorporated HindIII/XhoI restriction sites (Gene Synthesis, Invitrogen, Shanghai, China), and the fragment was subcloned to replace the 35S promoter in the pK7FWG2.0, yielding the proLIS-pK7FWG. Subsequently, LR reaction was conducted to make the proLIS-JsBEAT1-GFP and proLIS-JsBEAT2-GFP binary vectors, using the proLIS-pK7FWG and the respective pENTR/D-TOPO vectors harboring either *JsBEAT1* or *JsBEAT2* cDNA. These proLIS-driven expression vectors were used in transformation of *P.hybrida* W115.

All cloned sequences and constructs were verified by Sanger sequencing.

To generate sequence alignment and phylogenetic tree, the deduced amino acid sequences of *JsBEATs* and those of selected plant BAHD proteins previously characterized [15,19] were aligned by using Web-based Clustal and MAFFT programs with default parameters [38,39]. The MEGA X ver. 10.2.6 was used to analyze the phylogenetic relationship and to generate a Neighbor-Joining tree.

### 4.4. Recombinant Proteins and In Vitro Enzyme Reaction

The *JsBEAT2* cDNA were PCR amplified and subcloned into the pQE30 (QIAGEN) vectors, using *Bam*HI/*Pst*I restriction sites. The resulting plasmid was introduced into an *E. coli* M15 strain, and the transformant was grown to an 0.6 OD_600_ before induction of protein expression. The recombinant JsBEAT2 was best achieved by adding 0.4 mM isopropyl β-D-1-thiogalactopyranoside isopropyl β-d-1-thiogalactopyranoside (IPTG) and incubated for 10 h at 25 °C. Purification of the N-terminal-6xHis-tagged recombinant JsBEAT2 was carried out by using a Ni-NTA column kit (Sangon, Shanghai, China), with an elution buffer containing 80 mM imidazole.

Since *JsBEAT1* was not expressed well in the pQE30/M15 system, we reconstructed a pEGX-4T-1 (GE Healthcare) vector harboring the *JsBEAT1* cDNA at the *Bam*HI/*Not*I cloning sites. The *E. coli* Rosetta strain was used to express the N-terminal-GST-tagged JsBEAT1 protein. Culture of the recombinant Rosetta cells at an 0.6 OD_600_ was incubated for 16h at 25 °C under 0.4 mM IPTG to induce protein synthesis. Cell lysates were loaded on a GST4FF prepacked gravity column (Sangon, Shanghai, China), and the recombinant GST-JsBEAT1 was eluted with 30 mM glutathione-containing buffer.

All constructs were verified by sequencing, and protein concentration after purification was monitored via the Quick Start Bradford protein assay kits using bovine serum albumin as a standard (Bio-Rad). The expected size of the recombinant fusion proteins was checked by SDS-PAGE (Figure S5).

The enzyme activity was determined in a 320 μL reaction mixture consisting of 250 mM Tri-HCl (pH 7.5), 25 mM each of MgCl_2_ and KCl, 10 mM β-mercaptoethanol, and ~30 μg GST-JsBEAT1 or ~200 μg 6xHis-JsBEAT2 proteins, supplemented with varied concentrations of substrates (benzylalcohol and acetyl-CoA). The reaction was allowed for 1h at 30 °C and terminated by mixing with an equal volume of hexane containing 0.2 mM isobutylbenzene as an internal standard. The reaction mixture was filtered through a 0.22 μm membrane and subjected to GC-MS analysis. The optimization of the reaction conditions for pH and temperature were predetermined by using a set of appropriate buffers from pH 6.0 to 8.0 and by testing a range of 20 to 35 °C, with 1 mM acetyl-CoA/benzylalcohol as the substrates. All assays were performed in triplicate.

If not specific, all chemicals were purchased from Sigma-Aldrich.

### 4.5. Transient Expression and Stable Transformation

Protoplast isolation and PEG-mediated transfection using fresh *J. sambac* petals had been described previously [22]. The pK7-JsBEAT1 and pK7-JsBEAT2 vectors (Section 4.3) expressing C-terminus GFP-tagged proteins, as well as a pK7-GFP control vector expressing along the GFP were used to transfect the protoplasts. Transfected protoplasts were further incubated at 22 °C in the dark for 16–20h before observation under the confocal microscopy.

For leaf-infiltration, *A. tumefaciens* strain GV3101 harboring the respective pK7-based vectors was grown to an OD_600_ and resuspended in an infiltration medium (10 mM MES buffered MS medium containing 3% sucrose and 200 μm acetosyringone, pH 6.1). After 2–3 h incubation at room temperature, the agrobacterium suspension was used to infiltrate mature leaves of 4-week-old *N. benthamiana* plants. One day after the infiltration, the leaves were detached, and the epidermis was used for confocal microscopic observation.

To generated stable transgenic lines in *P. hybrida* cv ‘Mitchell Diploid’ (MD, or W115), *A. tumefaciens* strain GV3101 harboring the proLIS-JsBEAT1-GFP or proLIS-JsBEAT2-GFP vector (Section 4.3) was used to transform leaf explants following a standard method [40]. Transformant was selected on 100 mg L^−1^ kanamycin medium for regeneration. Regenerated plants derived from the T0 generation were further screened by PCR amplification of a *nptII* gene fragment. Transgenic plants among the progenies from T0 and subsequent T1 generations were selected by germinating the seeds on kanamycin-containing MS medium. Positive transgenic T2 generation was used for phenotypic examination, genetic verification (Figure S6) and floral volatile determination.

### 4.6. Confocal Fluorescence Microscopy

A Leica TCS SP8 confocal microscope (Leica Microsystems GmbH, Wetzlar, Germany) was used to monitor signals for GFP (excitation at 470/40 nm, emission at 164 525/50 nm) and for chlorophyll autofluorescence (excitation at 545/30 nm, emission at 620/60 nm) in specimens from protoplasts, leaf epidermis, and petals.

### 4.7. Statistical Analysis

Quantitative data were reported as mean ± SD (standard deviation) from three independent experiments or from three biological replications. When appropriate, significant difference between pair or among groups of treatments/samples was checked by T-test or ANOVA multiple tests using the software GraphPad Prism version 8.3.

### 4.8. Accession Number

The coding sequences have been submitted to NCBI GenBank under the accession numbers OK507197 for the *JsBEAT1* and OK507198 for *JsBEAT2*.

## Figures and Tables

**Table 1:** Major floral volatile benzenoids/phenylpropanoids in *J. sambac* petal extracts post anthesis.

Time	3-Hexenal	Concentration (mg g^−1^ FW) *					Benzoic Acid-2-Propenyl Ester
		Benzyl Alcohol	Benzyl Acetate	Methyl Anthranilate	cis-3-Hexenyl Benzoate	Benzyl Benzoate	
20:00	16.5 ± 1.5 a	41.3 ± 2.3 c	34.2 ± 1.7 e	10.7 ± 0.6 f	140.8 ± 1.3 d	14.3 ± 0.2 e	50.4 ± 8.4 c
23:00	15.3 ± 0.9 a	48.3 ± 3.5 b	199.4 ± 11.3 b	50.1 ± 3.1 d	208.9 ± 13.3 a	23.9 ± 1.0 c	69.0 ± 7.04 b
02:00	14.2 ± 1.1 a	48.3 ± 3.2 b	227.2 ± 10.0 a	87.2 ± 1. 7 b	228.2 ± 7.8 a	25.2 ± 0.62 b	77.6 ± 2.5 a
05:00	10.9 ± 0.6 c	55.4 ± 3.4 a	148.8 ± 5.9 c	95. 4 ± 3.4 a	170.2 ± 8.2 b	29.7 ± 1.1 a	80.9 ± 2.0 a
08:00	11.1 ± 0.7 bc	52.6 ± 1.2 a	75.9 ± 1.3 d	62.9 ± 2.9 c	134.8 ± 2.5 c	19.8 ± 0.6 d	42. 5± 0.6 d
11:00	12.5 ± 1.1 b	42.8 ± 0.3 c	33.4 ± 0. 8 ef	47.9 ± 1.1 d	68.7 ± 2.1 d	10.1 ± 0.2 fg	6.5 ± 0.2 e
14:00	7.7 ± 1.3 d	15.2 ± 1.2 d	27.8 ± 0.5 fg	26.6 ± 1.2 e	44.5 ± 3.6 e	8.9 ± 0.8 g	8.2 ± 1.0 e
17:00	2.3 ± 0.4 e	9.0 ± 0.2 e	24.1 ± 0.8 g	23.1 ± 1.7 e	13.8 ± 0.7 f	11.2 ± 1.1 f	7.5 ± 0.8 e

* Relative to an internal standard isobutylbenzene. Means ± SD followed by same letter indicates no significant difference within each column (*p* < 0.05, n = 3).

**Table 2:** Steady-state kinetic parameters of JsBEAT1 and JsBEAT2 summarized from Figure 4 and Figure 5.

		*K*_m_	*k*_cat_	*k*_cat_/*K*_m_
		μM	s^−1^	nM^−1^ s^−1^
GST-JsBEAT1	Benzyl alcohol (with acetyl-CoA)	447.3 ± 107.8	201.5 ± 4.1	450.5 ± 241.8
	Acetyl-CoA (with benzyl alcohol)	546.0 ± 94.5	316.8 ± 4.4	580.2 ± 173.6
6xHis-JsBEAT2	Benzyl alcohol (with acetyl-CoA)	278.7 ± 26.5	36.8 ± 0.6	132.0 ± 96.5
	Acetyl-CoA (with benzyl alcohol)	317.3 ± 31.7	25.7 ± 0.4	81.0 ± 67.4

## Data Availability

All data supporting the findings of this study are available within the paper and within its Supplementary Materials published online. Further information may be obtained from the corresponding author, B.W.

## Supplementary Materials

The following are available online at https://www.mdpi.com/article/10.3390/plants11010013/s1: Figure S1: Tentative identification of the floral volatile compounds with GS-MS based on the retention index (RI) and the MS spectrum comparing with that in the NIST11 (National Institute of Standards and Technology) GC-MS database; Figure S2: Expression profile of BAHD homologous genes in *J. sambac* transcriptomes; Figure S3: A Neighbor-Joining tree showing the phylogenetic relationship of the *J. sambac* BAHD-like proteins with known members from other plants; Figure S4: Amino acid sequence alignment of the 7 *J. sambac* BAHD-like proteins showing the highly conserved HXXXD and DFGWG motifs; Figure S5: Representative images showing subcellular localization of the C-terminus-tagged GFP fusion of JsBEAT1 or JsBEAT2.; Figure S6: Production of JsBEAT1 and JsBEAT2 recombinant proteins in *E. coli*; Figure S7: Generation and verification of transgenic *P. hybrida* plants ectopically expressing JsBEAT1-GFP and JsBEAT2-GFP driven by a floral-specific promoter from the *CbLIS* gene; Figure S8: Representative GC-MS chromatograms in petal hexane extracts of *P. hybrida* W115 and transgenic plants; Figure S9: Putative identification of floral volatile benzenoid/phenylpropanoid compounds in GC-MS analysis of *P. hybrida* corolla hexane extracts; Table S1: Prediction of subcellular localization of the JsBEATs; Table S2: List of primers used in this study.
