# Supplementary material for: Characterization of Two BAHD Acetyltransferases Highly Expressed in the Flowers of Jasminum sambac (L.) Aiton
Source: Plants (Basel). 2021 Dec 21;11(1):13. doi: 10.3390/plants11010013 (PMC8747370; doi:10.3390/plants11010013)
Supplement: Supplementary file 1 [file plants-11-00013-s001.zip › plants-1509910-supplementary.pdf]

plants

MDPI

Supplementary Materials for

Characterization of two BAHD acetyltransferases highly expressed in the flowers of *Jasminum sambac* (L.) Aiton

Yuting Wang *et al.*

Corresponding authors: Binghua Wu, binghua.wu@fafu.edu.cn

The PDF file includes:

Table S1 to S2

Figure S1 to S9

References

Table S1. Prediction of subcellular localization of the JsBEATs.

	CELLO2GO [a] (score at E-value < 0.001)	BUSCA [b] (score)	WoLF PSORT [c] (similarity)
JsBEAT1 (c163883)	cyt [d]: 1.229, P: 1.118, Pe: 1.015	cyt (0.7)	Pe: 9, Ch: 2, N: 1.5, cyt-N: 1.5, G: 1
JsBEAT2 (c142812)	cyt: 1.257, Ch: 0.889, M: 0.870	cyt (0.7)	cyt: 7, N: 4, M: 1, P: 1, cysk: 1
JsBEAT3 (c17043)	cyt: 1.431, Ch: 0.861	cyt (0.7)	M: 8, Ch: 6
JsBEAT4 (c162636)	P: 1.201, cyt: 1.169, Pe: 0.825	cyt (0.7)	cyt: 7, Ch: 3, N: 2, M: 2
JsBEAT5 (c79287)	Ch: 1.082, M: 0.962, Pe: 0.808	cyt (0.7)	cyt: 7, N: 2.5, Pe: 2, N-P: 2, Ch: 1, M: 1
JsBEAT6 (c12998)	cyt: 1.504, Ch: 1.003	cyt (0.7)	Ch: 7, cyt: 5, N: 1, M: 1
JsBEAT7 (c32665)	Ch:1.814, cyt: 0.817	C (1)	Ch: 10, N: 2, M: 2

Note: The three programs for prediction use different criteria.

[a] CELLO2GO, http://cello.life.nctu.edu.tw/cello2go/

[b] BUSCA, http://busca.biocomp.unibo.it/

[c] WoLF PSORT, https://wolfpsort.hgc.jp/

[d] Ch, chloroplast; cyt, cytoplasmic (or cytosolic); cyt-N, cytosolic-nucleus; cysk, cytoskeleton; G, Golgi apparatus; M, mitochondrial; N, nucleus; N-P, nucleus-plasma membrane; P, plasma membrane; Pe, peroxisome.

Table S2. List of primers used in this study.

Names	Sequences (5'-3')
For RT-qPCR	
q-JsBEAT1-F	CCATGTCCTTTCTTGGAGGA
q-JsBEAT1-R	CTCCAAAGGTAAAGCCACCA
q-JsBEAT2-F	TGTGGCTATGCTGGACAAAG
q-JsBEAT2-R	TAGCACTCTGGTGTCGGTTG
q-JsBEAT3-F	AGTGGCTGACACGGAAGAGT
q-JsBEAT3-R	CATCCCTTCTGTTGGGAAGA
q-JsBEAT4-F	AAGTCCAGCAAAACCCACAC
q-JsBEAT4-R	CTCTCGAATCACAGCCACAG
q-JsBEAT5-F	CAAGATTTCGGCTTTTCCAG
q-JsBEAT5-	CATGAACTCCTGGTCGGACT
q-JsBEAT6-F	AGGTTCAGCTGTGGCTCATT
q-JsBEAT6-R	ATGGTTCGGTCATGAAAAGG
q-JsBEAT7-F	TCGCGAGAGTCGATGATATG
q-JsBEAT7-R	ACTTGTGGGAACAACCTTGG
q-JsActin-F	TCTCTATGGTAACATTGTCCTG
q-JsActin-R	ATCCAGACACTGTACTTCCTCT
q-JsUBQ-F	GCTGGAAAGCAACTCGAA
q-JsUBQ-F	GGGGGATGCCTTCCTTATCT
For Gateway cloning	
TOPO-JsBEAT1-F	CACCATGGCAATTGCATTTAGAGTACACCGGCGTG
TOPO-JsBEAT1-R	TAACCTAGCACAACATTTTCTACTGGGAGATG
TOPO-JsBEAT2-F	CACCATGAGTCATTCAAACGGACGTGCAGCAG
TOPO-JsBEAT2-R	TTGATCGTAGTCCTTAATCAGCTTTTGGAC
Verif-M13-5F	GTAAAACGACGGCCAG
Verif-M13-3R	CAGGAAACAGCTATGAC
pK7-seq-35Spro-F	CAAATGCCATCATTGCGATA
pK7-seq-LISpro-F	TCTATAAATACAGATCCAGATCCACTTC
pK7-seq-GFP-R	AGCTTGCCGTAGGTGGCATCG
For *E.coli* expression vectors	
PGEX-*JsBEAT1*-F	GAGAGAGGATCCATGGCAATTGCATTTAGAGTACACCGGCGTG
PGEX-*JsBEAT1*-R	GAGAGAGCGGCCGCTAACCTAGCACAACATTTTCTACTGGGAGATG
PQE30-*JsBEAT2*-F	GAGAGAGGATCCATGAGTCATTCAAACGGACGTGCAGCAG
PQE30-*JsBEAT2*-R	GAGAGACTGCAGTTGATCGTAGTCCTTAATCAGCTTTTGGAC
For petunia transgenics	
Verif-Kan-5'	GCGTTCAAAAGTCGCCTAAG
Verif-Kan-3'	GCTCTTCGTCCAGATCATCC
q-PhUBQ-F	TGGAGGATGGAAGGACTTTGG
q-PhUBQ-R	CAGGACGACAACAAGCAACAG

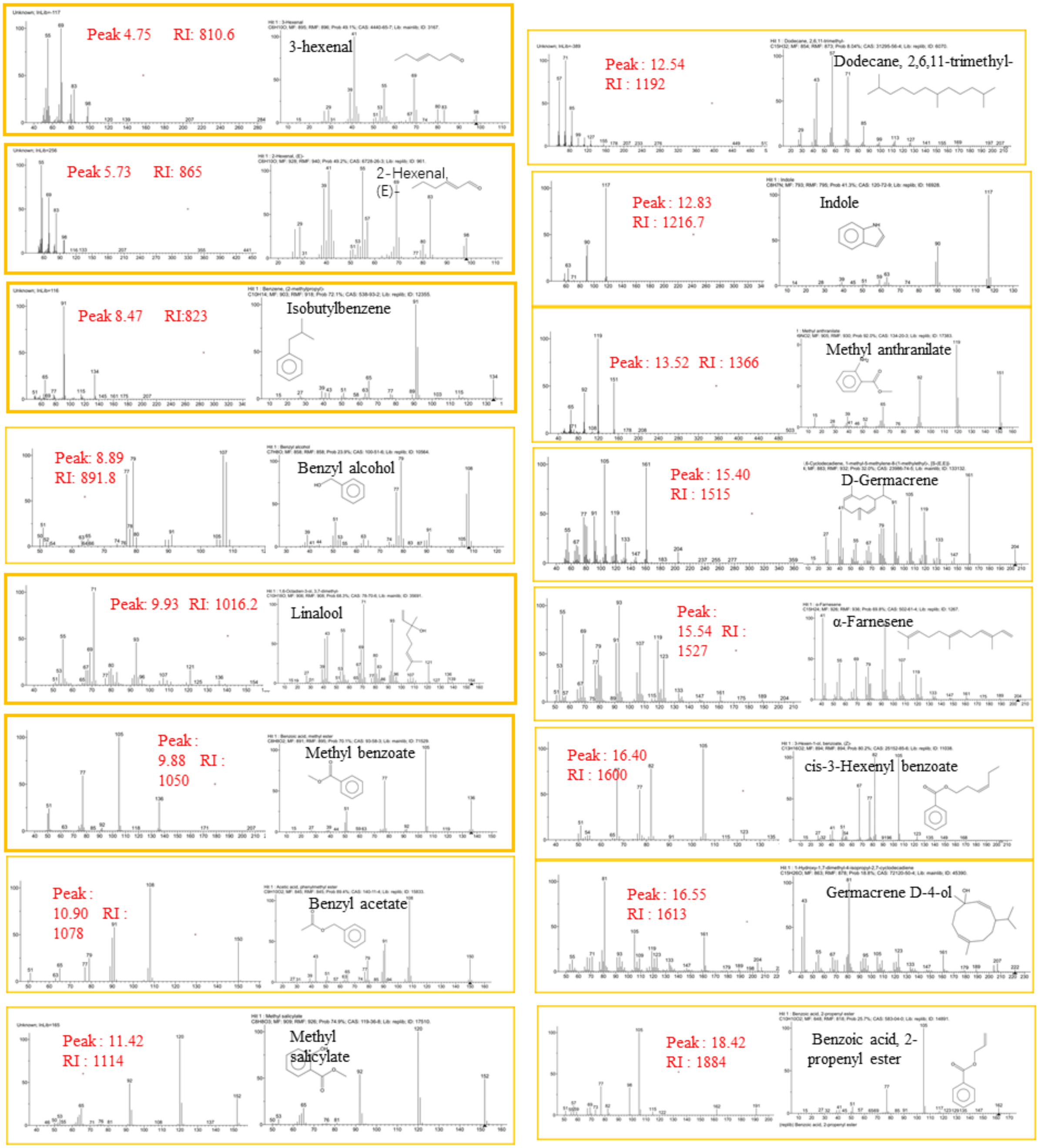

(Figure S1 continue to next page)

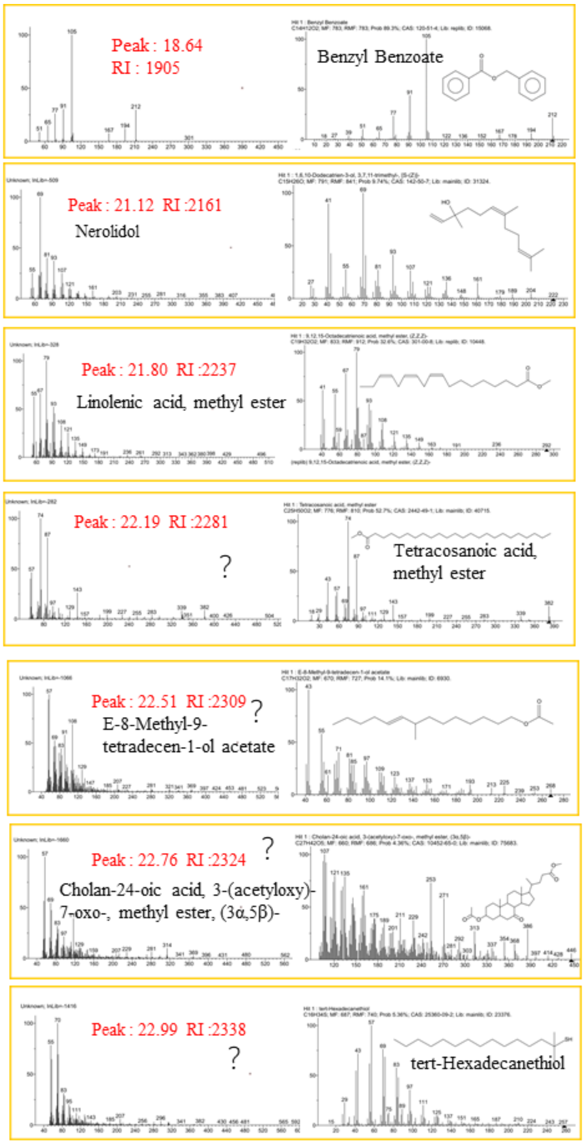

Figure S1. Tentative identification of the floral volatile compounds with GS-MS based on the retention index (RI) and the MS spectrum comparing with that in the NIST11 (National Institute of Standards and Technology) GC-MS database. Benzylalcohol, (R/S)-linalool, benzylacetate, α-farnesene, and benzylbenzoate were also confirmed using authentic referent compounds.

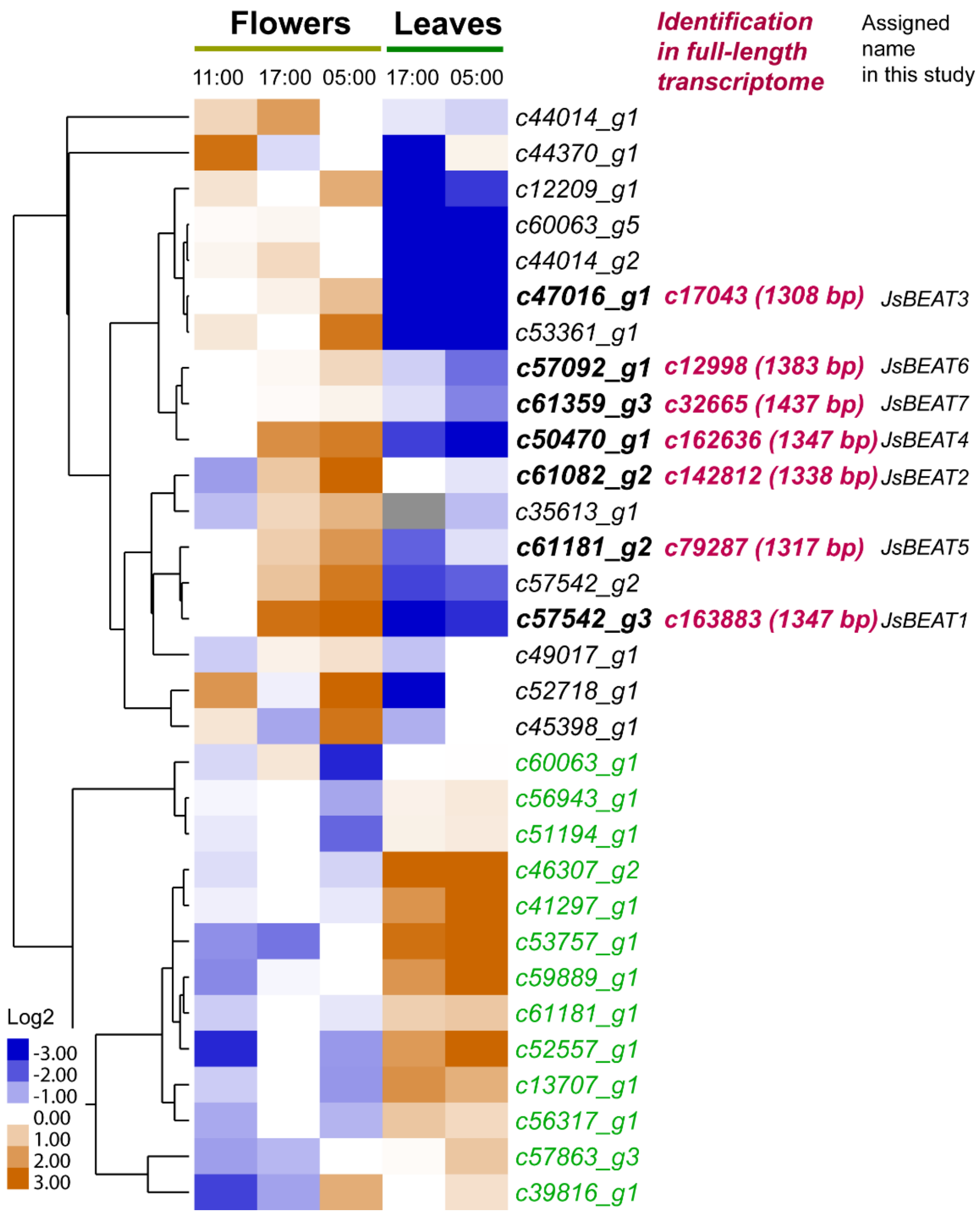

Figure S2. Expression profile of BAHD homologous genes in *J. sambac* transcriptomes. In addition to the curated unigenes (names in bold), this heatmap contains also unigenes with short (< 500 bp) and redundant sequences. A full-length PacBio library was blast with the unigenes to obtain the corresponding full-length sequences.

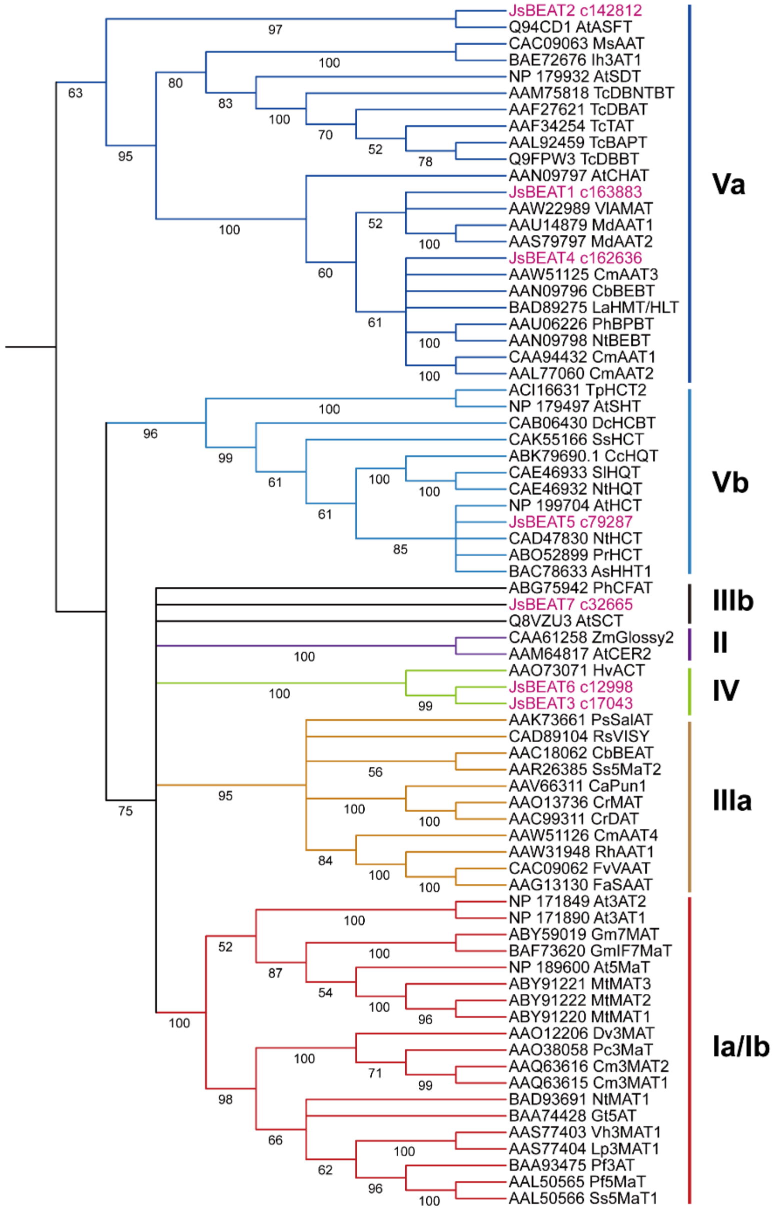

Figure S3. A Neighbor-Joining tree showing the phylogenetic relationship of the *J. sambac* BAHD-like proteins with known members from other plants. Representative protein sequences of previously characterized enzymes (the NCBI GenBank accessions and the enzyme names were shown in the tree) was retrieved from the NCBI/SwissPro databases and, sequence alignment was conducted using MAFFT [1] with default parameters. The alignment was further analyzed in MEGA X [2] with Bootstrap method (1000 replications). The Bootstrap numbers greater than 51 were shown under the branches.

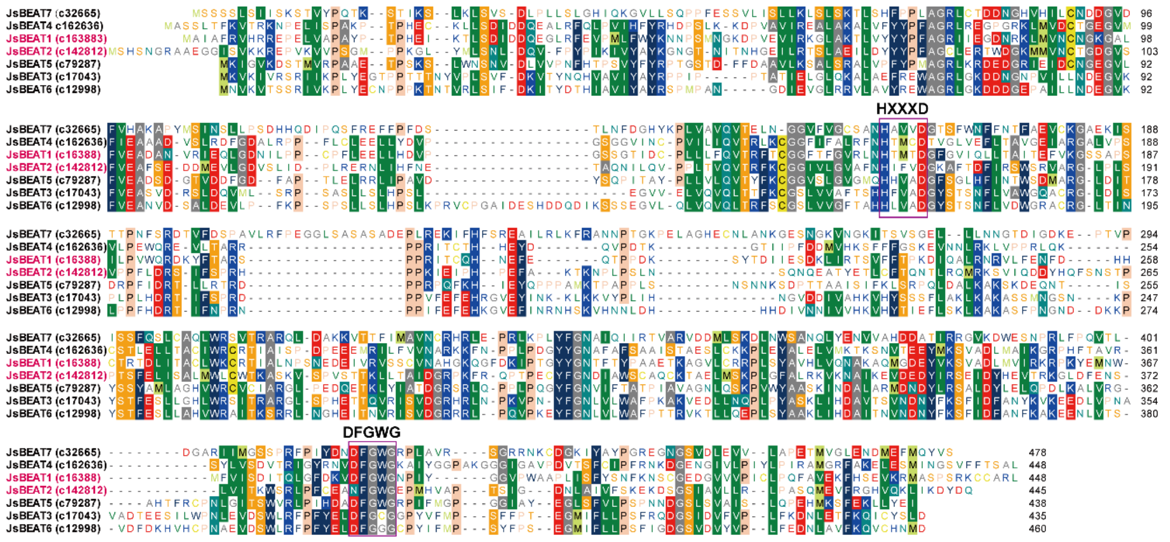

Figure S4. Amino acid sequence alignment of the 7 *J. sambac* BAHD-like proteins showing the highly conserved HXXXD and DFGWG motifs. The HXXXD was known for an important role in catalytic activity [3,4], while the DFGWG was likely involved in structural integrity [5].

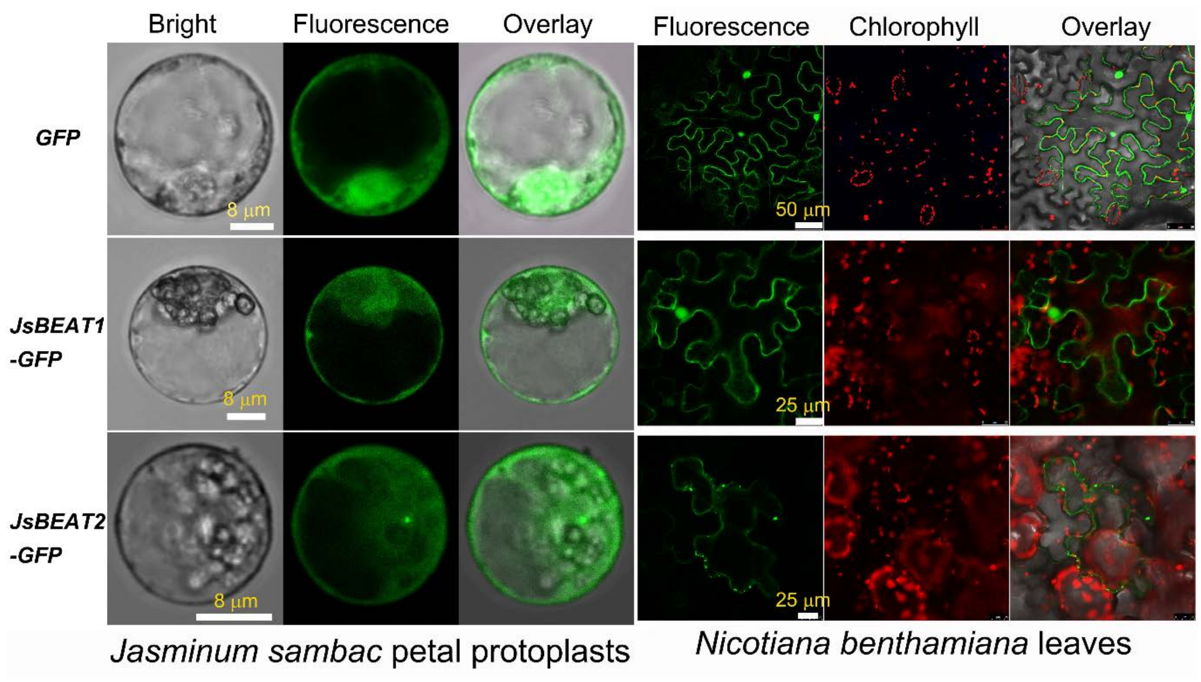

Figure S5. Representative images showing subcellular localization of the C-terminus-tagged GFP fusion of JsBEAT1 or JsBEAT2. Transient expression in petal protoplasts was conducted via PEG-mediated transfection of the respective constructs while *Agrobacterium* infiltration was used to introduce the expression the constructs in leaf epidermis. All constructs were driven by the 35S promoter from Cauliflower Mosaic Virus (CaMV).

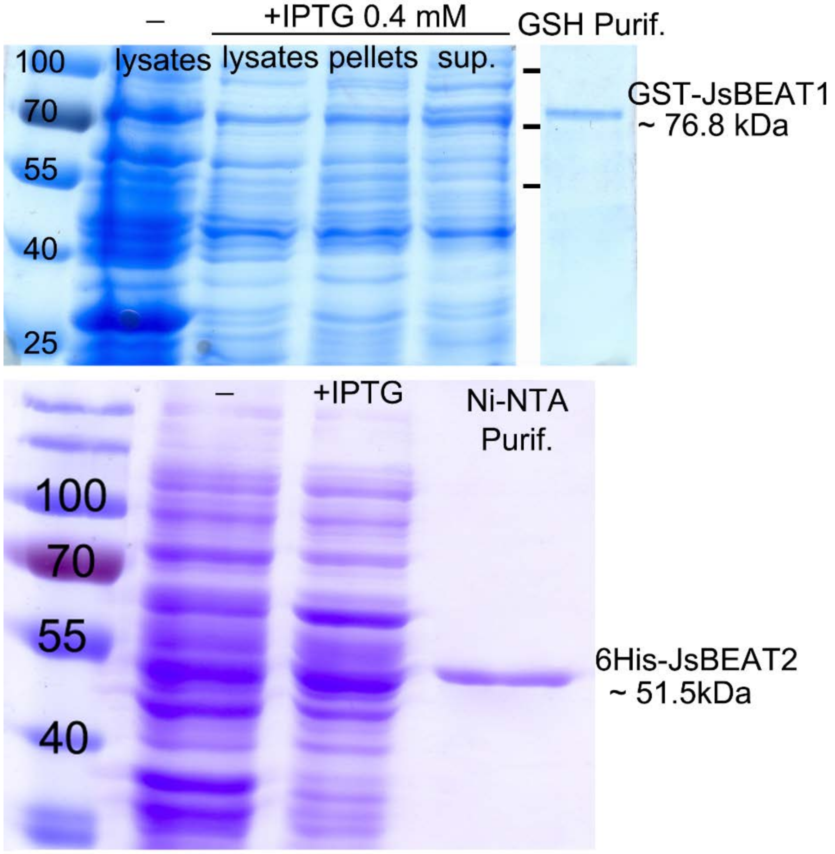

Figure S6. Production of JsBEAT1 and JsBEAT2 recombinant proteins in *E. coli*. Proteins were separated on 10% SDS-PAGE and the gel was stained with 1.25% Coomassie Blue R250.

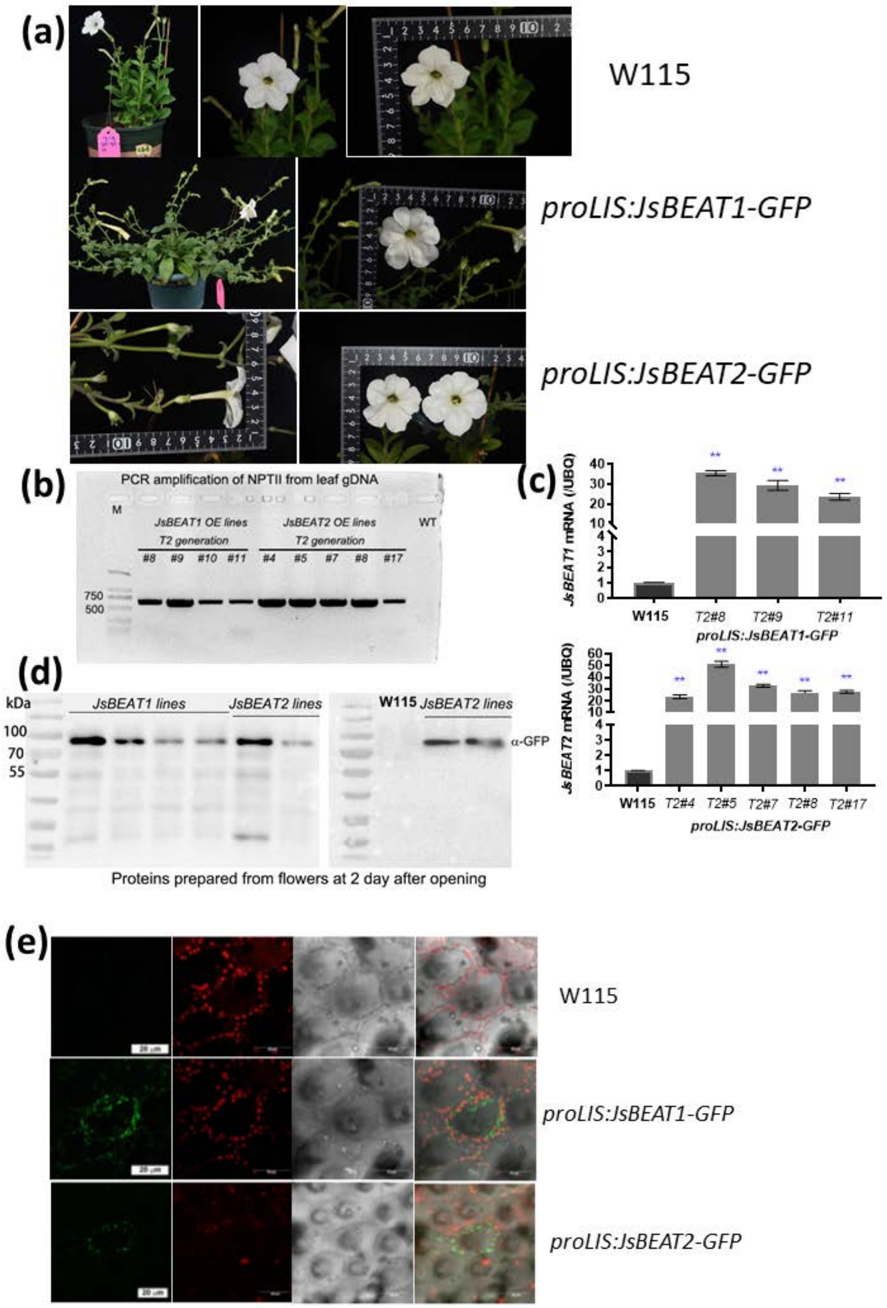

Figure S7. Generation and verification of transgenic *P.hybrida* W115 plants ectopically expressing *JsBEAT1-GFP* and *JsBEAT2-GFP* driven by a floral-specific promoter from the *CbLIS* gene. (**a**) Transgenic plants in the T2-generation showed normal flowering phenotype but with slightly more flowers; (**b**) PCR amplification of a 613-bp fragment of the *NPTII* gene in the T-DNA using gDNA preparation from leaf samples; (**c**) Quantitative RT-PCR determination of JsBEAT1 or JsBEAT2 transcript level in the corolla; (**d**) Immunodetection of GFP-fusion expressed in the flowers with the anti-GFP antibody; (**e**) Laser scanning confocal micrograph images showing the GFP florescence in the epidermal cells of the corolla in both transgenic plants, scale bar = 25 μm.

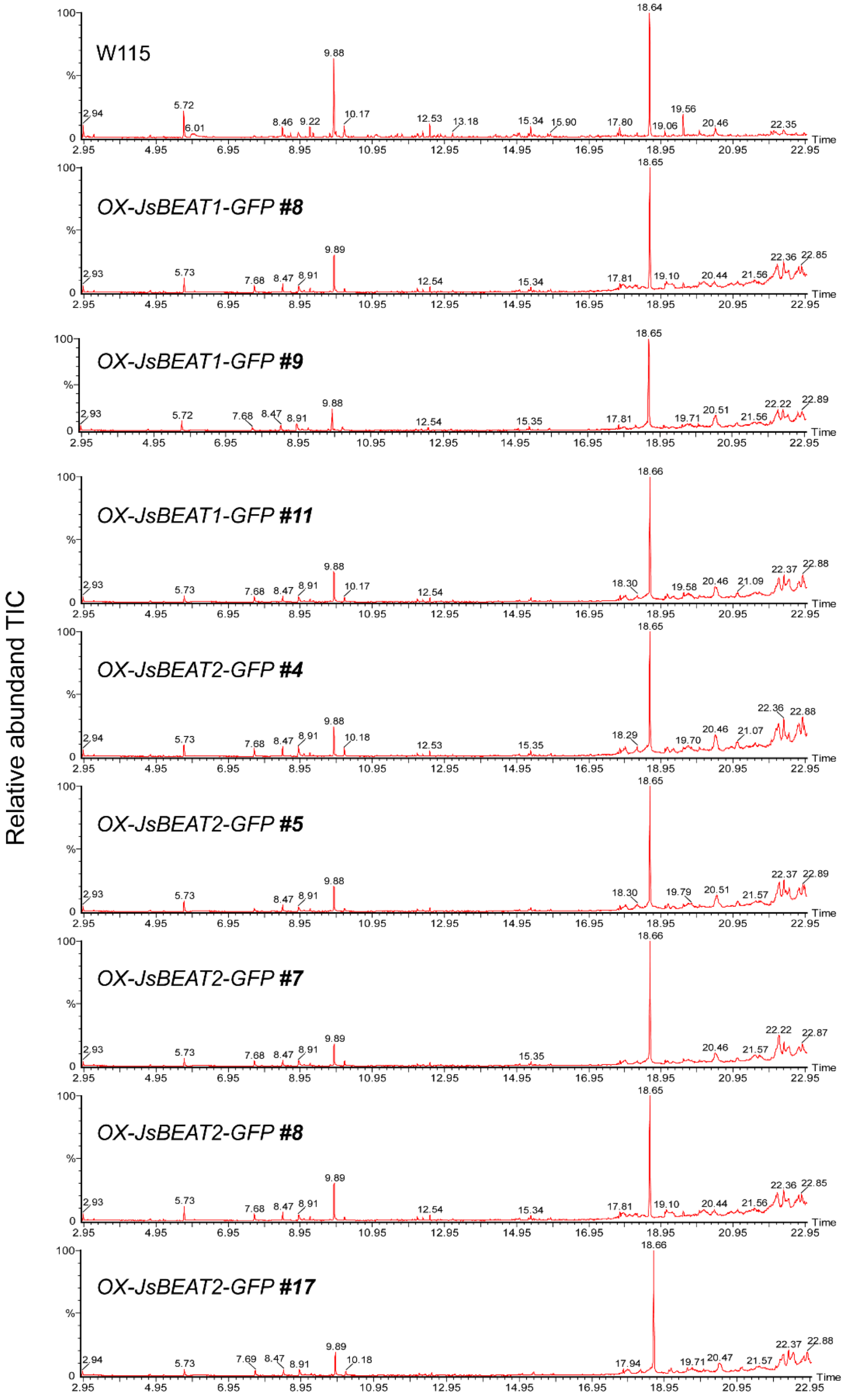

Figure S8. Representative GC-MS chromatograms in petal hexane extracts of *P.hybrida* W115 and transgenic plants. Independent lines were determined with three replications. Compound corresponding to each major peak was tentatively identified based on literatures and by matching with the RI and MS data.

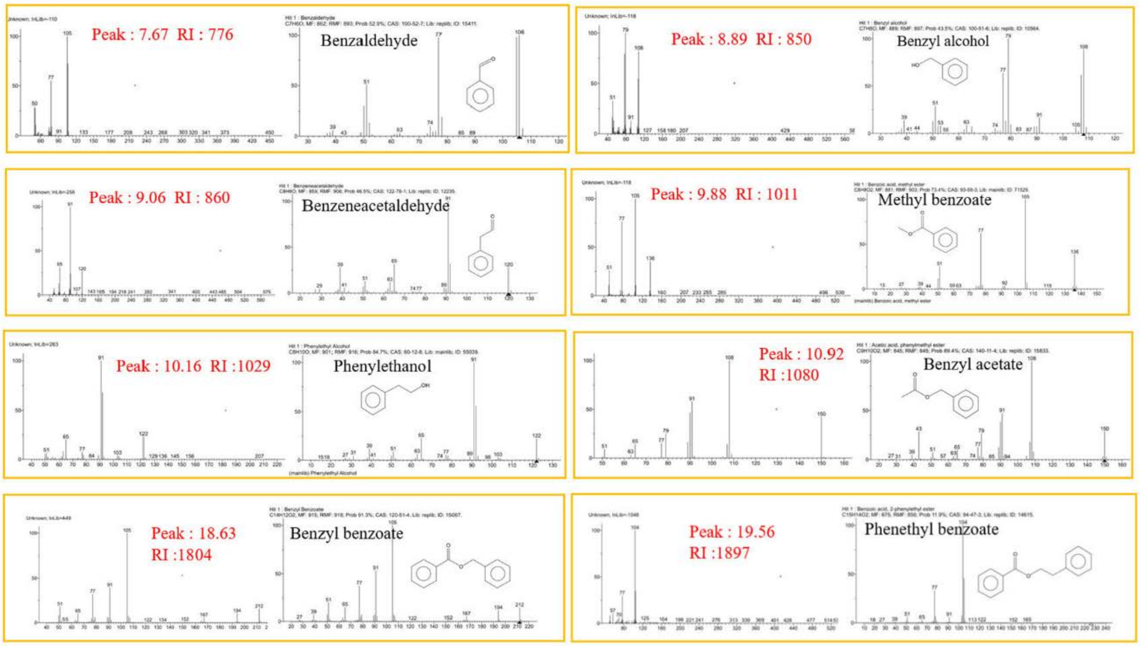

Figure S9. Putative identification of floral volatile benzenoid/phenylpropanoid compounds in GC-MS analysis of *P.hybrida* corolla hexane extracts (related to Figure S7).

References

1. Katoh, K.; Misawa, K.; Kuma, K.; Miyata, T. MAFFT: a novel method for rapid multiple sequence alignment based on fast Fourier transform. *Nucleic Acids Res* **2002**, *30*.
2. Kumar, S.; Stecher, G.; Li, M.; Knyaz, C.; Tamura, K. MEGA X: Molecular Evolutionary Genetics Analysis across Computing Platforms. *Molecular Biology and Evolution* **2018**, *35*, 1547-1549, doi:10.1093/molbev/msy096.
3. Molina, I.; Kosma, D. Role of HXXXD-motif/BAHD acyltransferases in the biosynthesis of extracellular lipids. *Plant Cell Reports* **2015**, *34*, 587-601, doi:10.1007/s00299-014-1721-5.
4. Bayer, A.; Ma, X.Y.; Stockigt, J. Acetyltransfer in natural product biosynthesis - functional cloning and molecular analysis of vinorine synthase. *Bioorganic & Medicinal Chemistry* **2004**, *12*, 2787-2795, doi:10.1016/j.bmc.2004.02.029.
5. Morales-Quintana, L.; Moya-Leon, M.A.; Herrera, R. Computational study enlightens the structural role of the alcohol acyltransferase DFGWG motif. *J Mol Model* **2015**, *21*, doi:ARTN 216 10.1007/s00894-015-2762-6.
